# Insecticide-treated durable wall lining for malaria control: multicentre studies from Africa and South-East Asia

**DOI:** 10.1186/1475-2875-11-S1-P121

**Published:** 2012-10-15

**Authors:** Louisa A Messenger, Abrahan Matias Arnez, JB Stiles-Ocran, Mamadou B Coulibaly, Marie-Louise Larsen, Nathan Miller, Adedapo O Adeogun, CEG Mulder, Hoan Le, Immo Kleinschmidt, Mark Rowland

**Affiliations:** 1 Department of Disease Control, London School of Hygiene and Tropical Medicine, London, UK; 2 Medical Care Development International (MCDI), Malabo, Equatorial Guinea; 3 Entomology Research Unit, Malaria Control Centre, AngloGold Ashanti Ltd., Obuasi, Ghana; 4 Vector Genomics and Proteomics, Malaria Research and Training Centre (MRTC), University of Sciences, Techniques and Technologies, Bamako, Mali; 5 Technical Institute of Denmark (DTU), Lyngby Denmark; 6 The MENTOR Initiative, Huambo, Angola; 7 Molecular Entomology and Vector Control Research Laboratory, Nigerian Institute of Medical Research, Lagos, Nigeria; 8 Agricultural Research Station (Pty) Ltd., Friedenhiem JT 282, Nelspruit, South Africa; 9 Vestergaard Frandsen Laboratories, 253/9 Minh Khai, Hai Ba Trung district, Hanoi, Vietnam

## Background

Indoor residual spraying (IRS) is a primary method of malaria vector control but its potential impact is constrained by several inherent limitations: spraying must be repeated when insecticide residues decay, householders may object to the annual imposition and campaign costs are recurrent. Durable wall lining (DL) can be considered a novel form of long-lasting IRS, which gradually releases insecticide over a period of three to four years when used to cover interior house walls. DL is designed to overcome the logistical constraints associated with repeated rounds of spraying whilst retaining the most attractive feature of IRS, the protection of all members of the community [1-3]. To establish DL as a viable substitute it must demonstrate equivalent or superior levels of bioefficacy, acceptability, durability and logistical feasibility to currently available products.

## Materials and methods

To identify a desirable material to develop into a durable wall lining, a one year preliminary trial was conducted among rural and urban households in Angola and Nigeria (n=258) comparing three deltamethrin-treated prototype materials (polyethylene shade cloth, laminated polyethylene sheeting and mosquito wall netting) [4]. The most popular lining material (shade cloth polyethylene, henceforth DL) was then evaluated in comparison with conventional IRS during a one year multicentre trial conducted in rural households in malaria endemic Equatorial Guinea, Ghana, Mali, South Africa and Vietnam (n=220).

## Results

During the preliminary trial a dichotomy between rural and urban participants emerged. Rural households favoured wall adornments and accepted wall linings because of their perceived decorative value and entomological efficacy, whereas urban households preferred minimal wall decoration and objected to the materials aesthetics and installation feasibility. Of the prototype lining materials assessed, polyethylene shade cloth DL was the most popular because of its ease of installation, aesthetics and resemblance to locally available materials. During the multicentre field trial, DL demonstrated consistently higher levels of bioefficacy compared to IRS, with no significant loss of bioactivity after 12 months. Field samples of DL retained on average 78% of their original insecticide content after one year. The majority of households reported reductions in mosquito density (93%) and biting (82%), but no adverse changes to their indoor environment (83%). When offered a choice of vector control product at the end of trial, the majority of participants chose DL regardless of the earlier household allocation.

## Conclusions

These two trials represent the largest field evaluation of DL to date [4]. The high level of acceptability among rural inhabitants identifies these communities as the ideal target consumer group for DL. DL remained fully efficacious against mosquito vectors, demonstrated minimal loss of insecticide content over 12 months of field use and was unequivocally more popular than IRS and other long-lasting vector control products. Together these results demonstrate that DL has the potential to overcome many of the operational challenges associated with IRS and may represent a viable long-lasting alternative, a scenario not dissimilar to the advantages and superiority shown by long-lasting insecticidal nets when introduced in place of conventional insecticide-treated nets.
